# Interleukin-35 Suppresses Antiviral Immune Response in Chronic Hepatitis B Virus Infection

**DOI:** 10.3389/fcimb.2017.00472

**Published:** 2017-11-13

**Authors:** Xue Shao, Jingting Ma, Shengnan Jia, Lanlan Yang, Wudong Wang, Zhenjing Jin

**Affiliations:** Department of Hepatopancreatobiliary Medicine, Second Hospital, Jilin University, Changchun, China

**Keywords:** hepatitis B virus, viral persistence, interleukin-35, CD8^+^ T cells, regulatory T cells

## Abstract

The mechanisms of hepatitis B virus (HBV) persistent infection are not completely understood. Interleukin (IL)-35, which is a newly identified cytokine belongs to IL-12 family, has been demonstrated to induce immunotolerance. Thus, the aim of current study was to investigate the role of IL-35 during chronic HBV infection. A total of 61 patients with chronic HBV infection [37 chronic hepatitis B (CHB) and 24 asymptomatic HBV carriers (ASC)] and 20 healthy individuals were enrolled. IL-35 concentration as well as the modulatory function of IL-35 on CD4^+^CD25^+^CD127^dim/−^ regulatory T cells (Tregs) and on HBV antigen-specific CD8^+^ T cells was investigated. IL-35 expression was significantly increased in both CHB and ASC, and was positively correlated with the levels of HBV DNA. Inhibition of viral replication induced the reduction in serum levels of IL-35. IL-35 stimulation led to inhibition of proinflammatory cytokine productions and elevation of apoptosis in peripheral blood mononuclear cells (PBMCs), but not in HepG2.2.15 cells. Moreover, IL-35 stimulation not only robustly inhibited cellular proliferation, but also up-regulated the production of IL-10 and IL-35 in a HBV antigen-specific and non-specific manner in Tregs/CD4^+^CD25^−^ T cells coculture system, which indicated enhancement of suppressive function of Tregs. Furthermore, IL-35 also reduced both cytolytic activity (direct lysis of HepG2.2.15 cells) and noncytolytic function (IFN-γ and TNF-α production) of HBV antigen-specific CD8^+^ T cells. The current data suggested that IL-35 contributed to maintain viral persistence by suppressing antiviral immune responses and reducing inflammatory responses in chronic HBV infection.

## Introduction

Hepatitis B virus (HBV) infection is still a worldwide public health problem with approximate 350 million chronic infections all over the world (Montuclard et al., 2015). Persistent HBV infection always results in end-stage liver diseases, including decompensated liver cirrhosis, liver failure, and hepatocellular carcinoma, leading to more than 1 million deaths annually owing to complications (Nannini and Sokal, 2017). The outcome of hepatitis B patients is closely associated with host immune status. Acute HBV infection in adults always induces a multispecific CD4^+^ T helper and CD8^+^ T cytotoxic responses with elevated interferon-γ (IFN-γ) production, which is important for viral clearance and controlling the infection (Sandhu et al., 2017). In contrast, cellular immune responses in chronic HBV infection are weak or undetectable, which lead to the collapse of HBV-specific adaptive immunity (Kennedy et al., 2017; Vyas et al., 2017). However, the precise mechanism of immune tolerance and hyporesponsiveness in chronic hepatitis B (CHB) is still not completely elucidated.

Interleukin (IL)-35 is a newly identified member of IL-12 cytokine family, and comprises two heterodimeric subunits, IL-12 α chain p35 and IL-27 β chain Epstein-Barr virus-induced gene 3 (EBI3) (Collison et al., 2007; Niedbala et al., 2007). IL-35 is one of the major effector cytokines which secreted by CD4^+^CD25^+^ regulatory T cells (Tregs), and plays important inhibitory function in both infectious and autoimmune diseases (Choi et al., 2015; Sawant et al., 2015; Guan et al., 2017). IL-35 was increasingly expression in gingival tissue and gingival crevicular fluid in chronic bacterial infection induced periodontitis (Mitani et al., 2015). Influenza A virus infection also induced enhancement of IL-35 in both peripheral blood mononuclear cells (PBMCs) and human primary lung cells (Chen et al., 2016; Wang et al., 2016). The elevation of IL-35 exhibited extensive antiviral activity against various viral infections (Wang et al., 2016).

Our recent study revealed that an immunosuppressive function of IL-35 in chronic hepatitis C virus (HCV) infection, and play contradictory roles in maintaining viral persistence and inhibiting inflammatory responses (Liu et al., 2017). Moreover, IL-35 was highly expressed in HBV antigen-specific CD4^+^ T cells and inhibited the function of HBV antigen-specific IFN-γ producing CD8^+^ T cells *in vitro* (Li et al., 2015). Thus, we hypothesized that IL-35 also contributes to immunotolerance in chronic HBV infection. To test this possibility, functional analyses for purified Tregs/CD8^+^ T cells from chronic HBV-infected patients were investigated in response to recombinant IL-35 stimulation *in vitro*.

## Materials and methods

### Subjects

A total of 61 patients with chronic HBV infection, including 37 patients with CHB and 24 asymptomatic HBV carriers (ASC), were enrolled in this study. All patients were hospitalized or followed up in The Second Hospital Jilin University from March 2014 to June 2015. The diagnoses of CHB and ASC were made in accordance with diagnostic standard of Chinese Guideline of Prevention and Treatment for CHB (2010 Version). No patients received antiviral or immunomodulatory therapy before baseline sampling. Patients who were co-infected with HIV or other hepatitis viruses, or afflicted with immune disorder or end-stage liver diseases were excluded from the study. All CHB patients received entecavir (ETV) therapy (0.5 mg 1/daily) after baseline sampling, and blood samples were also collected 48 weeks post-therapy. For normal controls, 20 of healthy individuals with matched age and sex ratio were also enrolled. The baseline characteristics of all enrolled subjects were shown in Table 1. The study conformed to the ethical guidelines of the 1975 Declaration of Helsinki. The protocol was approved by the Ethics Committee of The Second Hospital of Jilin University, and written informed consent was obtained from each participant.

**Table 1:** Baseline clinical characteristics of enrolled subjects.

	**NC**	**CHB**	**ASC**
Case	20	37	24
Sex (Male/Female)	13/7	24/13	15/9
Age (years)	30.1 ± 7.8	33.7 ± 10.1	26.9 ± 8.6
ALT (IU/L)	<40 (21.7 ± 8.4)	>80 (168.9 ± 59.7)	<40 (28.4 ± 6.7)
HBV DNA (log_10_ IU/mL)	Not available	5.94 ± 1.36	7.14 ± 1.07

*NC, normal control; CHB, chronic hepatitis B; ASC, asymptomatic HBV carriers*.

### Virological and biochemical assessments

Hepatitis B virus (HBV) DNA was quantified by a commercial real-time Polymerase Chain Reaction (PCR)-Fluorescence Quantitative Detection Kit for HBV DNA (DaAn Gene, Guangzhou, Guangdong Province, China) with the detection limit of 2 log10 IU/mL. Serum biochemical assessments were tested by Hitachi 7500 automatic analyzer (Hitachi, Tokyo, Japan).

### PBMCs isolation, CD8^+^ cells and CD4^+^CD25^+^CD127^dim/−^ cells purification

Peripheral blood mononuclear cells (PBMCs) were isolated by density gradient centrifugation using Ficoll-Hypaque (Sigma-Aldrich, St Louis, MO, USA). CD4^+^CD25^+^CD127^dim/−^ Tregs were purified using CD4^+^CD25^+^CD127^dim/−^ regulatory T cell isolation kit II (Miltenyi, Bergisch Gladbach, Germany). CD8^+^ T cells were purified using human CD8^+^ T cell isolation kit (Miltenyi). The purity of enrich cells was more than 90% by flow cytometry determination.

### Cell culture

HepG2.2.15 cells were cultured in Dulbecco's modified Eagle's medium (DMEM, Gibco, Grand Island, NY, USA) supplemented with 10% fetal bovine serum (FBS, Gibco), penicillin (100 U/L, Tiangen, Beijing, China), and streptomycin (0.1 mg/mL). Moreover, G148 (final concentration, 6.5 mg/mL) was added to the culture medium to maintain HepG2.2.15 cells. Cells were incubated at 37°C under 5% CO_2_ condition. CD8^+^ T cells purified from chronic HBV-infected patients who were positive for HLA-A2 were stimulated with recombinant human IL-35 (final concentration 1 ng/mL; Peprotech, Rocky Hill, NJ, USA) for 6 h. Cells were washed twice, and were co-cultured in direct contact and in parallel in indirect contact (effector and target cells were separated by a 0.4 μm membrane, which allowed the passage of soluble factors only) with target HepG2.2.15 cells (ratio of effector cells to target cells = 1: 5) in the presence of HBV core 18-27 epitope (HBc 18-27, sequence: FLPSDFFPSV, final concentration 10 μg/mL) for 48 h, as described previously (Phillips et al., 2010). The supernatants and target cells were harvested for further studies. CD4^+^CD25^+^CD127^dim/−^ Tregs were stimulated with recombinant human IL-35 (final concentration 1 ng/mL; Peprotech) for 6 h. Cells were washed twice, and were co-cultured in direct contact with autologus CD4^+^CD25^−^ cells at the ratio of 1: 4 in the presence of anti-CD3/anti-CD28 (final concentration, 1 μg/mL, eBioscience, San Diego, CA, USA) or recombinant HBV surface antigen (HBsAg, final concentration 10 μg/mL; AbD Serotec, Oxford, United Kingdom) for 48 h. The supernatants and cultured cells were harvested for further experiments.

### Enzyme linked immunosorbent assay (ELISA)

Concentration of IL-35 was measured by commercial ELISA kits (CUSABIO, Wuhan, Hubei Province, China) according to instructions from the manufacturer.

### Cytokine assay

The following cytokines levels in the cultured supernatants, including interferon (IFN)-γ, IL-1β, IL-10, IL-12p70, IL-6, IL-8, and tumor necrosis factor (TNF)-α, were tested by Human Proinflammation 7-Plex Base Kit (Meso Scale Discovery, Rockvillie, MD, USA) using SECTOR Imager (Meso Scale Discovery) following manufacturer's instructions.

### Flow cytometry

Peripheral blood mononuclear cells (PBMCs) or HepG2.2.15 cells were trypsinized, and were resuspened in 500 μL of Annexin V binding buffer. 5 μL of Annexin V-FITC (Beyotime Biotech, Wuhan, Hubei Province, China) and 5 μL of propidium iodide (PI, Beyotime Biotech) were added for 10 min incubation at room temperature in the dark. Cell apoptosis was analyzed with FACS Calibur analyzer (BD Biosciences), and were analyzed using FlowJo software version 8.6.2 (Tree Star, Ashland, OR, USA).

### Cellular proliferation assay

Cellular proliferation was determined by Cell Counting Kit-8 (CCK-8, Beyotime Biotech) according to instructions from the manufacturer.

### Western blot

Western blot analysis was performed as previously described (Liu et al., 2017). Briefly, total proteins were loaded and separated on SDS-PAGE gels, and were electroblotted onto PVDF membrane. The membrane was soaked for 2 h in a blocking solution (PBS containing 5% non-fat milk and 0.05% Tween 20) and incubated overnight in the presence of rabbit polyclonal antibodies targeting phosphorylated signal transducers and activators of transcription 1 (STAT1, phospho Y701, ab30645), STAT1 (ab31369), or mouse monoclonal anti-GAPDH (Abcam, 1:2,000 dilution). Horseradish peroxidase-conjugated goat anti-rabbit or goat anti-mouse antibody IgG (Abcam, 1: 2,000 dilution) was added to the membrane and incubated for an additional 2 h. Antigen-antibody complexes were observed using enhanced chemiluminescence (Western Blotting Luminol Reagent).

### Cytotoxicity of HepG2.2.15

The cytotoxicity of HepG2.2.15 cells was assessed by measuring the lactate dehydrogenase (LDH) expression in the supernatants at the end of each incubation period using LDH Cytotoxicity Assay Kit (Beyotime) according to the manufacturer's instructions.

### Statistical analysis

Statistical significance was determined by SNK-*q* test or paired *t*-test using SPSS version 19.0 for Windows (SPSS, Chicago, IL, USA). Values of *P* < 0.05 were considered as significant differences.

## Results

### IL-35 was increasingly expressed and correlated with viral replication in patients with chronic HBV infection

**Figure 1:**
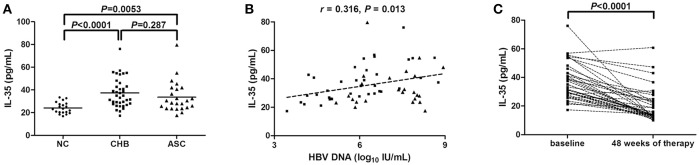
Interleukin (IL)-35 expression in patients with chronic hepatitis B virus (HBV) infection. **(A)** The concentration of IL-35 in the serum was measured by enzyme-linked imunosorbent assay (ELISA) in normal controls (NCs, *n* = 20), patients with chronic hepatitis B (CHB, *n* = 37), and asymptomatic HBV carriers (ASC, *n* = 24). Horizontal bars indicate mean value of each subset, and the individual level for each subject is shown. Significances were calculated using SNK-*q* test. **(B)** Pearson correlation analysis of IL-35 concentration with HBV DNA in 61 patients with chronic HBV infection (including CHB and ASC). **(C)** IL-35 concentration in the serum was also measured by ELISA in CHB patients receiving 48-week of entecavir therapy. The individual level for each subject is shown. Significance between baseline and 48 weeks post-therapy was calculated using paired *t*-test.

We firstly investigated IL-35 expression in the serum from all enrolled subjects, including 20 of normal controls (NC), 37 of CHB, and 24 of ASC. Serum concentration of IL-35 was significantly elevated in CHB (37.33 ± 12.72 pg/mL) and ASC (33.65 ± 13.64 pg/mL) in comparison with NC (24.17 ± 4.99 pg/mL; *P* < 0.0001 and *P* = 0.0053; Figure 1A). However, there was no remarkable difference of IL-35 level between CHB and ASC (*P* = 0.287; Figure 1A). Moreover, IL-35 expression was positively correlated with HBV DNA level in patients with chronic HBV infections (*r* = 0.316, *P* = 0.013; Figure 1B). However, there was no significant correlation between IL-35 concentration and serum ALT levels (*r* = 0.162, *P* = 0.615). Moreover, serum IL-35 levels were also measured in CHB patients receiving 48-week ETV therapy. ETV treatment led to significant down-regulation of HBV DNA levels, only one patient did not reach virological response at 48 weeks of therapy. Inhibition of viral replication resulted in remarkable reduction in IL-35 concentration in CHB patients (19.88 ± 11.40 pg/mL; *P* < 0.0001; Figure 1C).

### IL-35 stimulation decreased HBsAg-induced PBMCs proliferation and cytokine production

**Figure 2:**
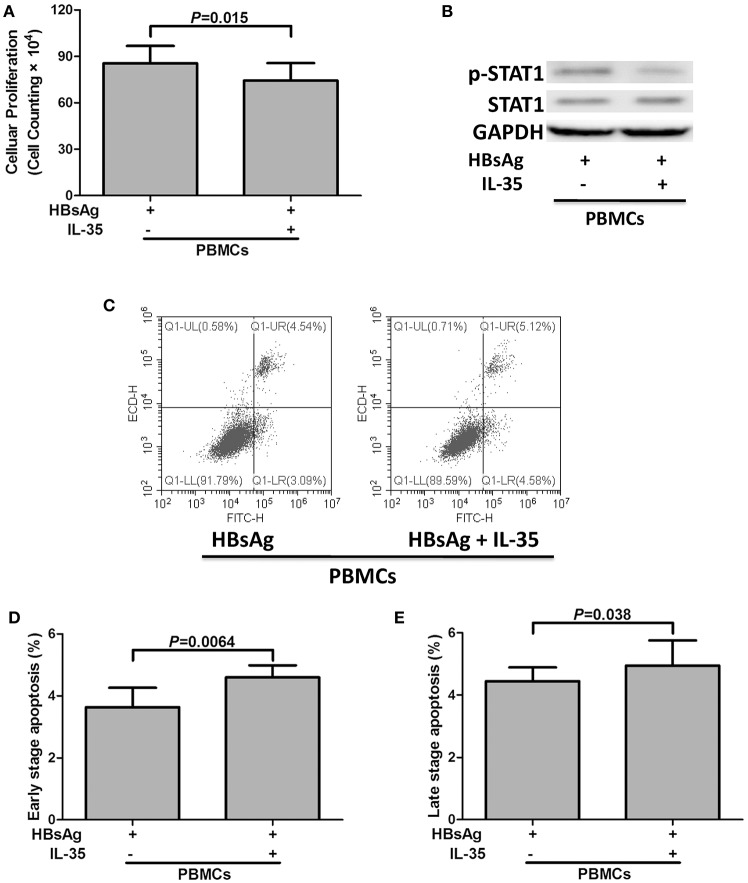
The regulatory role of interleukin (IL)-35 in peripheral blood mononuclear cells (PBMCs). PBMCs isolated from patients with chronic hepatitis B (CHB, *n* = 10) were stimulated recombinant hepatitis B surface antigen (HBsAg) in the presence or absence of recombinant IL-35 for 24 h. **(A)** Cellular proliferation was measured by cell counting kit-8. The data were presented as mean ± SD, and significances were calculated using paired *t*-test. **(B)** Phosphorylated STAT1 (p-STAT1) and total STAT1 were tested by Western blot, and GADPH was shown as control. **(C)** Representative flow cytometry analysis for apoptotic cells were shown in both PBMCs. Annexin V^+^PI^−^ cells (lower right) represented early stage apoptotic cells, while Annexin V^+^PI^+^ cells (upper right) represented late stage apoptotic cells. The percentages of early stage apoptotic cells **(D)** and late stage apoptotic cells **(E)** were shown. The data were presented as mean ± SD, and significances were calculated using paired *t*-test.

2 × 10^5^ of PBMCs isolated from 10 patients with CHB, which were randomly selected from the Figure 1A, were stimulated with HBsAg (10 μg/mL) in presence or absence of IL-35 (1 ng/mL) for 24 h. Cells and supernatants were harvested for further analyses. The results of CCK-8 assay showed that the growth of IL-35-treated PBMCs [cell counting, (7.43 ± 1.14) × 10^5^] were slower than those with HBsAg stimulation only [cell counting, (8.56 ± 1.12) × 10^5^; *P* = 0.015; Figure 2A]. The proinflammatory cytokine secretions in the cultured supernatants were also measured. The concentrations of IFN-γ, IL-1β, IL-6, and, IL-8, which were produced by PBMCs, were notably reduced in response to IL-35 stimulation (Table 2). In contrast, IL-10 level was remarkably elevated in HBsAg and IL-35 co-stimulated PBMCs in comparison with HBsAg stimulation only (Table 2). Reduction of proinflammatory cytokine secretion in HBsAg and IL-35 co-stimulated PBMCs was accompanied by the decreased phosphorylation of STAT1 in comparison of HBsAg stimulation only (Figure 2B). Furthermore, flow cytometry was also performed to assess the apoptotic cells. Representative Annexin V/PI stained PBMCs for apoptosis analysis were shown in Figure 2C. Annexin V^+^PI^−^ cells represented early stage apoptotic cells, while Annexin V^+^PI^+^ cells represented late stage apoptotic cells. IL-35 stimulation led to significant elevation in both early and late stage apoptotic PBMCs (*P* = 0.0064 and *P* = 0.038, respectively, Figures 2D,E).

**Table 2:** Cytokine production by PBMCs in response to IL-35 stimulation.

	**HBsAg**	**HBsAg+IL-35**	*P*-value^*^
IFN-γ (pg/mL)	76.40 ± 21.55	55.49 ± 23.68	0.0091
IL-1β (pg/mL)	10.68 ± 7.57	8.73 ± 6.86	0.017
IL-10 (pg/mL)	9.24 ± 2.98	15.52 ± 3.82	0.0019
IL-12p70 (pg/mL)	26.21 ± 21.42	20.06 ± 14.93	0.419
IL-6 (pg/mL)	54.55 ± 23.49	42.83 ± 20.11	0.036
IL-8 (pg/mL)	14.01 ± 7.02	8.63 ± 3.03	0.026
TNF-α (pg/mL)	176.5 ± 97.21	138.8 ± 29.96	0.247

* *Paired t-test was used for comparison between two groups*.

### IL-35 stimulation did not affect bioactivities of HepG2.2.15 cells

**Figure 3:**
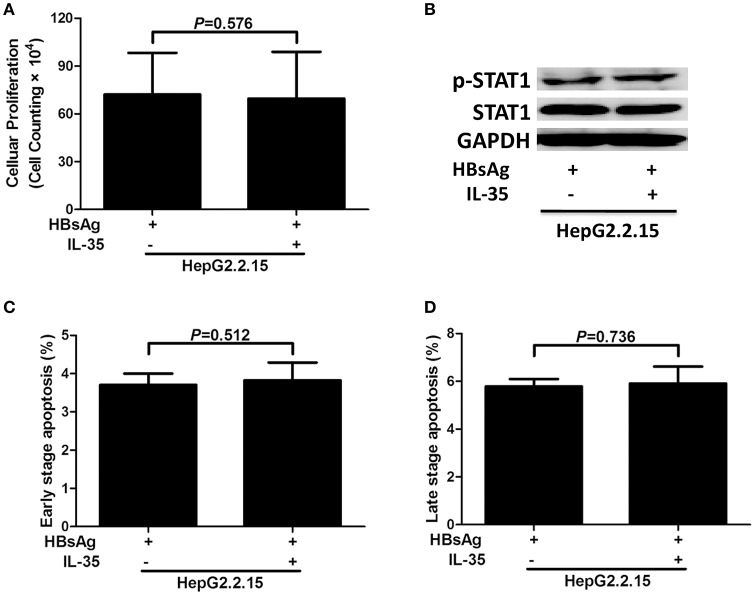
The regulatory role of interleukin (IL)-35 in HepG2.2.15 cells. HepG2.2.15 cells were stimulated recombinant hepatitis B surface antigen (HBsAg) in the presence or absence of recombinant IL-35 for 24 h, and were performed independently for five times. **(A)** Cellular proliferation was measured by cell counting kit-8. The data were presented as mean ± SD, and significances were calculated using paired *t*-test. **(B)** Phosphorylated STAT1 (p-STAT1) and total STAT1 were tested by Western blot, and GADPH was shown as control. The percentages of early stage apoptotic cells **(C)** and late stage apoptotic cells **(D)** were shown. The data were presented as mean ± SD, and significances were calculated using paired *t*-test.

5 × 10^4^ of HepG2.2.15 cells (seeded in five independent wells) were stimulated with HBsAg (10 μg/mL) in presence or absence of IL-35 (1 ng/mL) for 24 h. Cells and supernatants were harvested for further analyses. Cell proliferation did not change significantly in HBsAg-stimulated HepG2.2.15 cells in response to IL-35 treatment [(7.22 ± 2.62) × 10^5^ vs. (6.96 ± 2.93) × 10^5^; *P* = 0.576; Figure 3A]. Neither IL-10 nor IL-12p70 could be detected in the supernatants from HepG2.2.15 cells, and the production of other five cytokines also did not reveal significant differences in response to IL-35 treatment (Table 3). Phosphorylated STAT1 expression in HBsAg-stimulated HepG2.2.15 cells did not change significantly in the presence or absence of IL-35 (Figure 3B). Furthermore, there were no remarkable differences in percentage of apoptotic HepG2.2.15 cells between HBsAg and HBsAg+IL-35 stimulation (*P* > 0.05, Figures 3C,D).

**Table 3:** Cytokine production by HepG2.2.15 cells in response to IL-35 stimulation.

	**HBsAg**	**HBsAg+IL-35**	*P*-value^*^
IFN-γ (pg/mL)	38.51 ± 7.79	35.53 ± 14.29	0.686
IL-1β (pg/mL)	6.28 ± 2.13	5.25 ± 1.44	0.058
IL-10 (pg/mL)	N.D.	N.D.	—
IL-12p70 (pg/mL)	N.D.	N.D.	—
IL-6 (pg/mL)	52.08 ± 20.47	55.25 ± 24.01	0.854
IL-8 (pg/mL)	8.65 ± 3.08	7.70 ± 2.34	0.500
TNF-α (pg/mL)	352.5 ± 103.4	230.5 ± 64.12	0.655

* *Paired t-test was used for comparison between two groups*.

### IL-35 stimulation enhanced the inhibitory function of tregs in patients with chronic HBV infection

**Figure 4:**
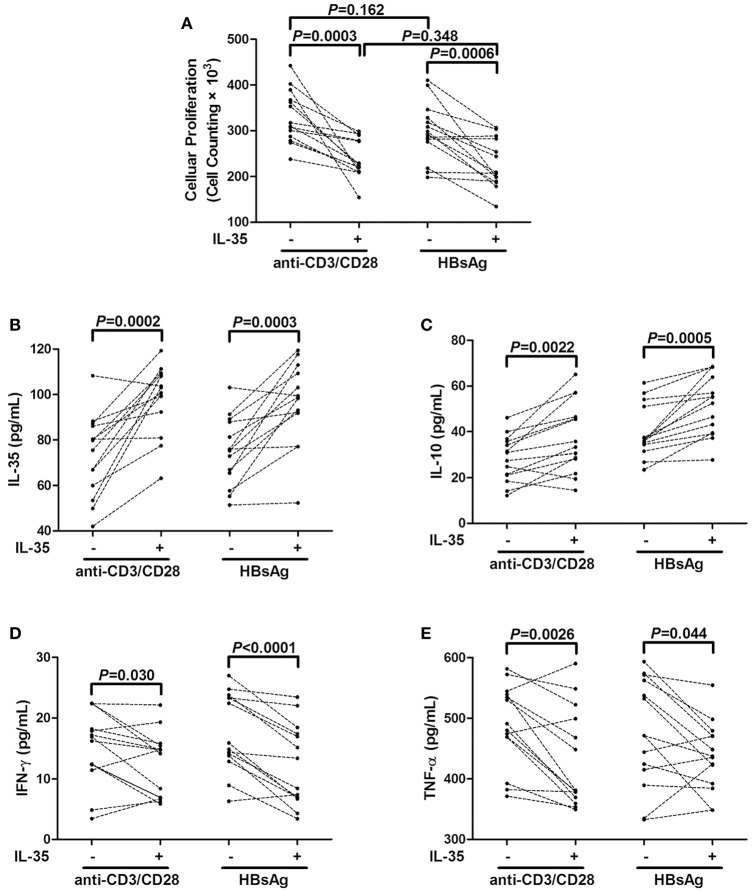
Interleukin (IL)-35 stimulation enhanced suppressive function of CD4^+^CD25^+^CD127^dim/−^ regulatory T cells (Tregs) from patients with chronic hepatitis B (CHB, *n* = 14). Purified CD4^+^CD25^+^CD127^dim/−^ Tregs and autologous CD4^+^CD25^−^ T cells were co-cultured at the ratio of 1: 4 in the presence or absence of IL-35 with either anti-CD3/CD28 or recombinant hepatitis B surface antigen (HBsAg) stimulation. **(A)** Cellular proliferation was measured by cell counting kit-8. Levels of cytokines in the supernatants of co-cultured system was also measured. **(B)** IL-35 concentration was measured by enzyme-linked imunosorbent assay. Levels of IL-10 **(C)**, interferon-γ (IFN-γ) **(D)**, and tumor necrosis factor-α (TNF-α) **(E)** were measured by Human Proinflammation 7-Plex Base Kit using SECTOR Imager. The individual level for each subject is shown. Significances were calculated using paired *t*-test.

A total of 2.5 × 10^4^ purified CD4^+^CD25^+^CD127^dim/−^ Tregs from 14 CHB patients, which were also randomly selected from Figure 1A, were stimulated with IL-35 for 6 h, and cells were washed twice with DMEM to remove recombinant IL-35. Stimulated CD4^+^CD25^+^CD127^dim/−^ Tregs were co-cultured with autologous CD4^+^CD25^−^ T cells at ratio of 1: 4 in either a non-specific (anti-CD3/CD28 stimulation) or HBV antigen specific (HBsAg stimulation) manner. Cells and medium supernatants were harvested after another 48 h of culture. It was mainly CD4^+^ T cells which produced the cytokines since the co-culture of CD4^+^CD25^+^CD127^dim/−^ Tregs and CD4^+^CD25^−^ T cells. There were no remarkable differences in the suppressive capacities of purified Tregs between anti-CD3/CD28 and HBsAg stimulation in the absence of IL-35 stimulation (*P* = 0.160, Figure 4A). IL-35 treatment notably increased the inhibitory activity of Tregs in both groups, which manifested as down-regulation of cellular proliferation (*P* = 0.0003 and *P* = 0.0006, respectively, Figure 4A). The enhancement effect represented similar potent in co-cultures between the two groups (*P* = 0.348, Figure 4A). Moreover, the levels of IL-35, IL-10, IFN-γ, and TNF-α were measured in the supernatants of co-cultured cells. IL-35 treatment enhanced the production of IL-35 and IL-10 in both non-specific and HBV antigen-specific cultures (Figures 4B,C). In contrast, IFN-γ and TNF-α secretions was reduced in response to IL-35 treatment in co-cultures subjected to either anti-CD3/CD28 or HBsAg stimulation (Figures 4D,E).

### IL-35 stimulation inhibited both cytolytic and noncytolytic function of CD8^+^ T cells in chronic HBV infection

**Figure 5:**
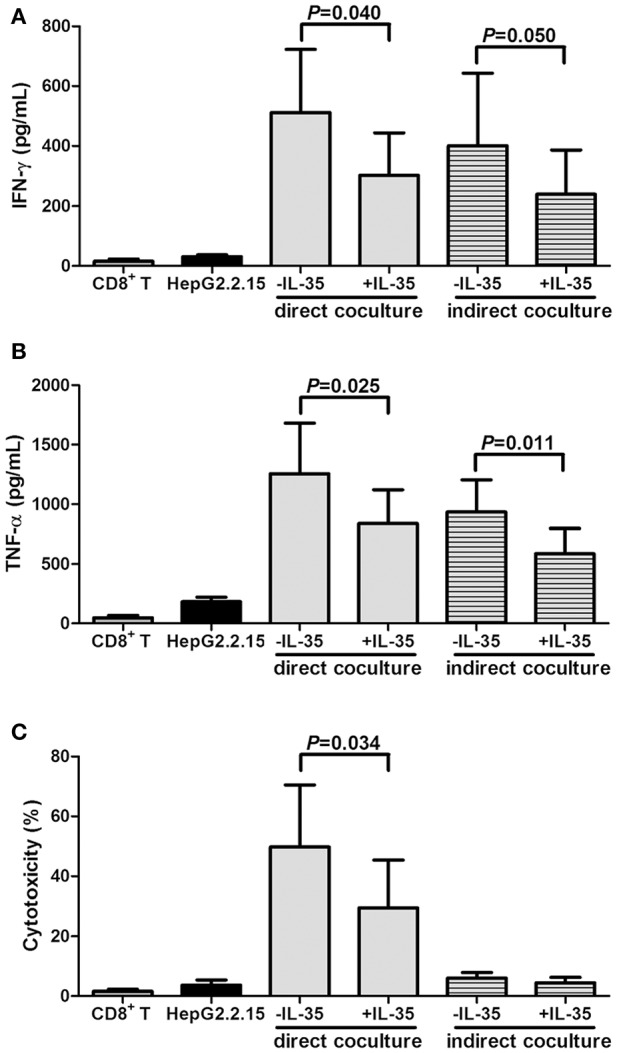
Interleukin (IL)-35 stimulation suppressed cytolytic and noncytolytic function of CD8^+^ T cells in chronic hepatitis B (CHB, *n* = 9). Purified CD8^+^ T cells from HLA-A2 restricted CHB patients were co-culture with HepG2.2.15 cells in the presence or absence of IL-35 with HBc 18-27 peptide stimulation in either direct or indirect contact culture system. As controls, HepG2.2.15 cells were cultured alone, and CD8^+^ T cells were stimulated with HBc 18–27 peptide. The concentrations of interferon-γ (IFN-γ) **(A)** and tumor necrosis factor-α (TNF-α) **(B)** in the supernatants were measured by Human Proinflammation 7-Plex Base Kit using SECTOR Imager. **(C)** Percentage of cell death was measured by lactate dehydrogenase (LDH) release. The data were presented as mean ± SD, and significances were calculated using paired *t*-test.

2 × 10^5^ of purified CD8^+^ T cells from HLA-A2 restricted CHB patients (*n* = 9) were stimulated with IL-35 for 6 h, and were co-cultured in direct contact or in indirect contact with 10^6^ of HepG2.2.15 cells in the presence of HBc 18-27 peptide. As controls, HepG2.2.15 cells were cultured alone, and CD8^+^ T cells were cultured with HBc 18-27 peptide stimulation only. The supernatants were harvested 48 h post co-culture for further analysis. IFN-γ and TNF-α productions in the supernatants were significantly increased in the direct and indirect coculture system (Figures 5A,B). As expected, IL-35 treatment down-regulated both cytokines secretion in the direct and indirect coculture system (*P* < 0.05, Figures 5A,B). Moreover, the cytotoxicity of target HepG2.2.15 cells were assessed after direct and indirect contact with CD8^+^ T cells, and percentage of cell death was measured by LDH release. A maximum of nearly 50% cell death was reach in direct contact coculture system, and IL-35 stimulation reduced percentage of cell death to approximate 30% (*P* = 0.034, Figure 5C). However, no cytotoxicity was observed in the indirect system with or without IL-35 stimulation, as the proportion of cell death was similar to cultured HepG2.2.15 cells (Figure 5C).

## Discussion

In the present study, we observed that the elevated serum IL-35 in chronic HBV-infected patients (both CHB and ASC) was positively correlated with HBV DNA level, whereas effective anti-HBV therapy down-regulated IL-35 expression, indicating a close relationship between IL-35 and HBV viral replication. Furthermore, IL-35-induced enhancement of Treg activity was found in both HBV antigen-specific and non-specific manner. Meanwhile, IL-35 also revealed significant immunosuppressive activities to HBV antigen-specific CD8^+^ T cells in both cytolytic and noncytolytic manner. The current results suggested that IL-35 regulated the functions of viral specific Tregs and CD8^+^ T cells during chronic HBV infection, which might be contribute to immunotolerance and viral persistence.

IL-35 is a recently identified heterodimeric cytokine, which is composed of IL-12p35 and EBI3 (Collison et al., 2007; Niedbala et al., 2007). Due to lack of commercial antibody to IL-35 several years ago, previous study showed the increase in both mRNA and protein level of EBI3 in patients with hepatitis B associated liver cirrhosis, which indicated the involvement of IL-35 in the pathogenesis of HBV related liver diseases (Shi M. et al., 2015). However, it was insufficient for supporting the issue that HBV infection induced IL-35 elevation. Liu et al. revealed that IL-35 could only be detected in CD4^+^ T cells isolated from CHB patients but not in those from healthy individuals by immunoprecipitation plus Western blot analysis using anti-EBI3 and anti-IL-12p35 antibodies (Liu et al., 2011). Moreover, Zhou et al. demonstrated that both IL-35 mRNA in CD4^+^ T cells and IL-35 protein expression in the serum was significantly higher in chronic HBV infection, especially in patients with high viral load (Zhou et al., 2015), which was consistent with the our present results. We also found a direct relationship between IL-35 expression and HBV replication, which manifested as the correlation between IL-35 elevation and HBV viral load. However, IL-35 did not directly impact on HBV-infected hepatocytes, since cellular proliferation, apoptosis, and STAT1 phosphorylation did not change in response to IL-35 stimulation in HepG2.2.15 cells, and no remarkable correlation was found between IL-35 and ALT level. Although it has been well accepted that IL-35 was a potential immunosuppressive and anti-inflammatory cytokine (Banchereau et al., 2012; Bobryshev et al., 2015; Xiang and Xie, 2015), *in vivo* studies also showed that IL-35 mediated and promoted inflammation in sepsis (Cao et al., 2015) and rheumatoid arthritis (Thiolat et al., 2014; Filkova et al., 2015). Thus, the role of IL-35 in chronic HBV infection needs to be further elucidated.

CD4^+^CD25^+^CD127^dim/−^ Tregs activities presented the escape mechanisms responsible for virus-induced immune regulation, which appeared to cause chronic infections and viral persistence in HBV infection (Zhang et al., 2010). IL-35 has been demonstrated as one of the major effector cytokines secreted by Tregs (Collison et al., 2007). It was also well established that IL-35 suppressed the proliferation and function of effector T cells. Our previous study also expanded the regulatory function of IL-35 in CD4^+^CD25^+^CD127^dim/−^ Tregs during chronic HCV infection (Liu et al., 2017). Shi et al. also revealed a positive correlation between IL-35 and FoxP3 mRNA expression in CHB patients (Shi Y. Y. et al., 2015). Thus, it was possible that the elevated proportion of Tregs (Zhang et al., 2010) might be the major source of IL-35 enhancement in the serum of patients with CHB. Herein, we also found that IL-35 stimulation increased the inhibitory function of CD4^+^CD25^+^CD127^dim/−^ Tregs by reducing cellular proliferation and enhancing IL-35/IL-10 productions. The augmentation of suppressive function induced by IL-35 stimulation was similar in both HBV antigen-specific and non-specific manner. The current results suggested that Tregs might directly responded to IL-35 stimulation, while IL-35 might also exert and positive feedback mechanism to enhance its own production (Sawant et al., 2015; Ma et al., 2016). Moreover, HBV antigen specific proinflammatory cytokines (IFN-γ and TNF-α) productions were reduced in response to IL-35 in both cultured PBMCs and Treg/CD4^+^CD25^−^ T cells coculture system. This indicated an anti-inflammatory activity of IL-35 in CHB, although we did not observe notable correlation between IL-35 and ALT level. Thus, the immunosuppressive property of IL-35 could sustain Tregs function and is likely to contribute to HBV persistence.

Both viral escape mutations and T cells exhaustion contributed to the failure in viral clearance in chronic HBV and HCV infection (Wieland et al., 2017). CHB often showed weak or absent virus-specific CD8^+^ T cells response, which presented as exhaustion state characterized by poor cytotoxic activity, impaired cytokine production, and expression of multiple inhibitory receptors (Ye et al., 2015). Li et al. demonstrated that IL-35 suppressed the proliferation of HBV antigen-specific cytotoxic T lymphocytes and IFN-γ secretion *in vitro* (Li et al., 2015). IFN-γ was also Th1 secreting cytokine, which contributed not only to liver cell injury, but also to recovery from disease and successful control of infection (Penna et al., 1997). However, CD8^+^ T cells control of HBV replication involved both cytolytic and cytokine-mediated noncytolytic mechanisms (Phillips et al., 2010). Thus, the *in vitro* direct and indirect contact coculture systems used in this study allowed us to investigate independently the cytolytic and noncytolytic functions of HBc 18-27 (an HLA-A2-restricted human immunodominant epitope) specific CD8^+^ T cells purified from HLA-A2 restricted CHB patients. Viral-specific CD8^+^ T cells could not only kill HBV-infected HepG2.2.15 cells, but also purge HBV infection from HepG2.2.15 cells, which mediated by IFN-γ and TNF-α production without inducing cellular damage. IFN-γ and TNF-α were two major cytokines to mediate CD8^+^ T cell antiviral activity (Guidotti et al., 1999; Li et al., 2016). IL-35 stimulation down-regulated both IFN-γ and TNF-α productions in both direct and indirect coculture systems, indicating IL-35 inhibited cytokines-induced antiviral immunity to HBV. Moreover, IL-35 also suppressed HBV antigen-specific cytotoxic CD8^+^ T cells in direct coculture system. However, no significant cytotoxicity was found between HepG2.2.15 cells in indirect contact with CD8^+^ T cells and HepG2.2.15 cells cultured alone, indicating cytokine produced by CD8^+^ T cells did not have a cytotoxic effect on target cells.

In summary, we found that HBV-induced elevation of IL-35 expression might potentiate the inhibitory function of CD4^+^CD25^+^CD127^dim/−^ Tregs, reduce both cytolytic and noncytolytic activities of HBV antigen-specific CD8^+^ T cells, and down-regulate expression of proinflammatory cytokines. The current data suggested that IL-35 contributed to maintain viral persistence by suppressing antiviral immune responses and reducing inflammatory responses in chronic HBV infection.

## Author contributions

XS, JM, and SJ performed the study. XS, LY, WW, and ZJ enrolled the patients. XS, JM, LY, WW, and ZJ analyzed the data, and prepared the manuscript. ZJ designed and supervised the study.

### Conflict of interest statement

The authors declare that the research was conducted in the absence of any commercial or financial relationships that could be construed as a potential conflict of interest.
